# Evolving threats: Leveraging *C. elegans* to decode the virulence profiles of highly related environmental *Salmonella* Newport isolates

**DOI:** 10.1371/journal.pone.0343882

**Published:** 2026-03-02

**Authors:** Christina M. Ferreira, Mirae Choe, Bella Wayhs, Julie A. Haendiges, Robert Literman, Jianghong Meng, Arjuman Ghazi, Rebecca L. Bell

**Affiliations:** 1 Office of Applied Microbiology and Technology, Office of Laboratory Operations and Applied Science, Human Foods Program, United States Food and Drug Administration, College Park, Maryland, United States of America; 2 Department of Nutrition and Food Science, University of Maryland – College Park, College Park, Maryland, United States of America; 3 Departments of Pediatrics and Cell Biology and Physiology, University of Pittsburgh School of Medicine; John G. Rangos Sr. Research Center, One Children's Hospital Drive, Pittsburgh, Pennsylvania, United States of America; 4 Division of Surveillance and Data Integration, Office of Surveillance Strategy & Risk Prioritization, Human Foods Program, United States Food and Drug Administration, College Park, Maryland, United States of America; University of Graz, AUSTRIA

## Abstract

*Salmonella enterica* subspecies *enterica*, particularly serovar Newport, remains a leading cause of foodborne illnesses in the United States, implicated in numerous outbreaks associated with a diverse array of food products. This study systematically investigates the virulence of five distinct *S*. Newport isolates, characterized by varying patterns of pulse-field gel electrophoresis (PFGE) molecular-diagnostic subtyping, using the nematode *Caenorhabditis elegans* as a host model organism. We conducted viability assays on *C. elegans* to evaluate how these isolates affect nematode survival. The selected bacterial strains, chosen for their historical significance in foodborne outbreaks yet isolated form environmental sources, were previously sequenced to provide a comprehensive genomic framework. A notable focus of our research was on the nearly genetically identical PFGE types Newport-61 and the Newport-1015 isolates, which differ by a 1.7 Mb genomic inversion. *C. elegans* survival assays in response to pathogenic-strain infections revealed that one Newport-1015 and the Newport-61 isolates were particularly more virulent compared to other isolates tested. These findings enhance our understanding of the pathogenic potential of environmental *S*. Newport and highlight the need to understand the regulatory mechanisms that contribute to virulence capacity.

## Introduction

*Salmonella enterica* subspecies *enterica* is the leading cause of microbial foodborne illnesses in the United States, resulting in over 1 million infections and more than 400 deaths each year [1,2]. Among its serovars, *S. enterica* serovar Newport (*S*. Newport) ranks as the second most common culture-confirmed serovar associated with foodborne outbreaks in the country [3]. *S.* Newport has a broad host range, implicated in illnesses linked to a variety of foods, including tomatoes, cucumbers, papaya, onions, and ground beef [4–9]. From 2000 to 2020, there were 227 foodborne salmonellosis outbreaks attributed to *S.* Newport, with six of those being recurrent outbreaks over a 12-year period connected to tomatoes from the Virginia Eastern Shore (VES) [4,10,11]. These tomato outbreaks were linked to a clonal strain of *S.* Newport, identified through PFGE as “Pattern 61” (*XbaI* JJPX01.0061, Newport-61). This clone is now categorized by the CDC under REPJJP03, which also includes *S*. Newport Pattern 1015 (*XbaI* JJPX01.1015, Newport-1015).

The nematode *Caenorhabditis elegans* has been established as a valuable model organism for studying the virulence mechanisms of human pathogens. A short lifespan (2–3 weeks) along with remarkable amenability to molecular, genetic and physiological interventions has led to discovery of fundamental innate immune mechanisms relevant to human infectious diseases [12,13]. Despite the absence of dedicated immune cells, worms deploy highly conserved immune response pathways such as the p38 MAPK response and TGF-beta pathways that have been studied extensively [14,15]. While *C. elegans* lacks the nuances of innate immunity present in mammalian immune systems, similarities in detecting infections and their resulting immune responses renders this model organism invaluable for studying pathogens that higher eukaryotes and humans are susceptible to as well as anti-microbial drug discovery [16,17]. Numerous pathogens, including *S*. enterica, colonize the host worm through their intestine following consumption and hence it is especially suitable for modeling immune mechanisms elicited by food-borne pathogens. Previously, worms exposed to *S. enterica* serovar Typhimurium (Typhimurium) strains were shown to have a significantly shortened lifespan with visible signs of infection in the intestinal tract. Notably, *Salmonella* strains that exhibit reduced virulence in mammals were shown to have similarly attenuated impact on the lifespan of *C. elegans* [18,19]. This indicates that bacterial genes important for pathogenic potency in vertebrate hosts are likely similarly required during *C. elegans* infection, highlighting its value to assess pathogenic virulence and host-pathogen interactions.

Newport-61 and Newport-1015 have been isolated from the surface water and sediment of the VES, with a seasonal recurrence of clonal isolates [20,21]. However, there are few documented cases, prior to the retirement of CDC PFGE PulseNet, of Newport-1015 causing human infection, suggesting overall less virulence capacity. In this study, we used *C. elegans* to evaluate the virulence of five *S*. Newport environmental isolates, each representing different PFGE patterns of historical significance related to foods. These isolates are closely related, with Newport-1015 and Newport-61 being particularly noteworthy, as they are nearly genetically identical except for a 1.7 Mb inversion [22].

## Materials and methods

### Bacterial isolates

Strains used in the *C. elegans* experiment included *Escherichia coli* OP50 (negative control) and six *Salmonella enterica* strains found in Table 1. The *S.* Typhimurium isolate (CFSAN000741) was used as the positive control for the *C. elegans* assays, and the *S.* Newport isolates (CFSAN000859, CFSAN001461, CFSAN001891, CFSAN003353, and CFSAN001469) were previously reported [22]. All strains were maintained as frozen stocks in Brain Heart Infusion Broth (BHIB, BD Difco) with 50% glycerol (v/v), and experiments were conducted with freshly grown cultures, streaked from frozen stock cultures onto Luria Bertani agar (LBA, BD Difco) and incubated at 35°C ± 2°C for 22 ± 2 hours [23]. Single colonies from these plates were inoculated into 3mL of Luria Bertani broth (LB, BD Difco) and incubated statically at 35°C ± 2°C for 22 ± 2 hours.

**Table 1:** *Salmonella* strains used for bioinformatic analysis and *C. elegans* assays.

Sample ID	Serovar	Source	Sequence Type	PFGE Pattern	Genome Accession Number
CFSAN001891	Newport	Goose feces	118	JJPX01.1015	CP140739 [22]
CFSAN001461	Newport	Sediment	118	JJPX01.1015	CP140738 [22]
CFSAN003353	Newport	Sediment	118	JJPX01.0061	CP140737 [22]
CFSAN001469	Newport	Sediment	350	JJPX01.0044	CP140735 [22]
CFSAN000859	Newport	Stool	5	JJPX01.0011	CP140750 [22]
CFSAN000741	Typhimurium	Tissue	--	--	JBREBA000000000

### *Salmonella* WGS comparative analysis

Strains used in this study were previously sequenced to provide complete, reference quality genomes using the PacBio [24]. The assemblies were uploaded to NCBI and annotated using the NCBI Prokaryotic Genome Annotation Pipeline (PGAP, v. 6.10) [25]. Roary (v. 3.13.0) was used to generate a pangenome alignment and to identify genes that were unique to the different PFGE patterns [26]. Visualization of the output from Roary were created using Phandango (v. 1.3.1) [27]. AMRFinderPlus (v. 4.0.3, with default settings and “plus genes”) and Phastest (v. 3.0 with deep annotation) were used to identify antimicrobial resistance (AMR), virulence, and phage genes [28–30]. BLASTN, hosted by NCBI with default settings, was used to identify the percent identity of the strains [31]. Geneious Prime (v 2025.1.2) was used for *in silico* restriction digestion of the strains with the enzyme *XbaI* to identify the difference in PFGE pattern comparison of JJPX01.0061 and JJPX01.1015.

### *Caenorhabditis elegans* survival assay

*C. elegans* survival experiments were conducted at 20°C with the wild-type strain, N2, using standard techniques. Worms were maintained on nematode growth medium (NGM), a standard, nutrient-rich solid media used for *C. elegans* culture as described previously [32,33]. Briefly, NGM plates were seeded with 70 μL of the worms’ normal laboratory diet of *E. coli* strain OP50 (OP50) or one of the six *Salmonella* pathogenic strains. OP50 plates were allowed to dry for 24 hours before adding worms. *Salmonella*-seeded plates were used after 5 hours of drying; hence fresh *Salmonella* plates were prepared every 24 hours.

For the survival assays, healthy, gravid young adult worms were transferred to fresh OP50-seeded NGM plates, allowed to lay eggs for 1–2 days and removed. The eggs were reared at 20°C and age-matched L4-stage, pre-adult larvae were selected for the experiment and transferred to NGM plates seeded with either the OP50 control bacteria or various *Salmonella* strains. Thirty L4-stage worms were transferred to each plate, with a total of 150 worms tested per bacterial strain. Worm survival was scored twice daily by gently touching the head, tail, or midsection with a platinum wire pick; worms were considered dead if they failed to respond. Worms were transferred to fresh, corresponding plates every 24 hours. Survival monitoring continued until all original worms died, using 12-hour/12-hour or 8-hour/16-hour scoring intervals. Worms that exploded, bagged, crawled off the plate, or were otherwise unaccounted for were censored from analysis. All survival assays were conducted twice in two biological replicates. Survival statistics were plotted using the Kaplan–Meier method. Statistics were calculated using the nonparametric log-rank Mantel−Cox method on the OASIS2 platform (v.2.4.2) and subjected to multiplicity Bonferroni correction [34].

## Results and discussion

### Genomic analysis reveals a large-scale inversion distinguishing Newport-61 and Newport-1015 lineages

**Fig 1:**
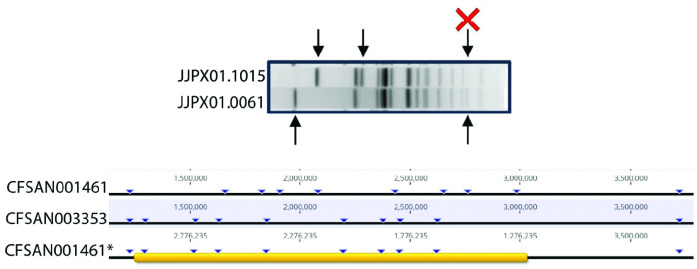
Phylogeny showing gene presence/absence of key Salmonella Newport isolates. The phylogenetic tree shows the relatedness of the S. Newport isolates to one another. The blue colored area represents the presence/absence of genes across the entirety of the genomes. The sequence types represented include 118 (magenta), 350 (yellow) and 5 (dark blue).

**Fig 2:**
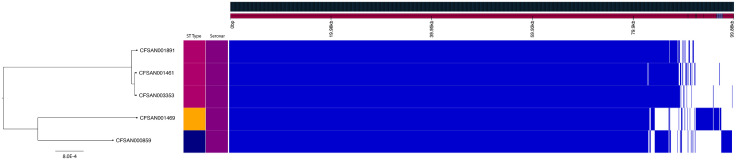
Inversion confirmation of CFSAN001461 and CFSAN1891 using in silico PFGE analysis. PFGE *XbaI* pattern of JJPX01.1015 compared to JJPX01.0061 (top), where the arrows highlight the differing bands in the PFGE patterns and the red “X” denotes the band that is not present in the 1015. The inversion identified in JJPX01.1015 accounts for the differences in the PFGE patterns due to changes in cut site locations within the genome. The locations of these cut site changes (bottom) due to the directionality of the inversion – by manually rotating the 1.7Mbp JJPX01.1015 inversion at the Gifsy-1 sites (denoted by CFSAN001461*) and subsequently performing *in silico* digestion with *XbaI*, the cut sites revert to the same fragment sizes reflected in JJPX01.0061 (CFSAN003353) confirming the accuracy of inversion in the closed genome.

The strains presented in this study are all known to cause human infections to varying degrees and have been identified across the United States. Gene content analysis shows that these isolates demonstrate over 98% genetic identity to one another, with Newport-61 being more than 99% identical to Newport-1015 (Fig 1). Analysis of genetic content related to virulence, AMR or prophage presence did not show any differences among the S. Newport isolates in this study (Table 2 and S1 Table). It was previously reported that two strains, CFSAN001891 and CFSAN001461 (both JJPX01.1015), have a 1.7 Mbp inversion relative to the chromosome of CFSAN003353 (JJPX01.0061) [22]. As can be seen in Fig 1, the 1.7 Mbp inversion accounts for the difference in PFGE patterns between JJPX01.0061 and JJPX01.1015 as the location of the XbaI restriction enzyme site is altered (Fig 2, indicated by “X”).

**Table 2:** AMRFinder Plus identified virulence and antimicrobial resistance genes for *Salmonella* isolates in this study.

Gene	Description
*allD*	ureidoglycolate dehydrogenase AllD
*avrA*	type III secretion system YopJ family effector AvrA
*envF*	putative envelope lipoprotein EnvF
*fieF*	CDF family cation-efflux transporter FieF
*fljA*	phase 1 flagellin gene repressor FljA
*gip*	hydroxypyruvate isomerase Gip
*gogB*	type III secretion effector GogB
*golS*	Au(I) sensor transcriptional regulator GolS
*golT*	gold/copper-translocating P-type ATPase GolT
gtgA*	type III secretion system effector protease GtgA
gtrC1*	putative glucosyltransferase GtrC1
*hscC*	molecular chaperone HscC
*icmF*	type VI secretion protein IcmF
*int*	FimB-specific integrase
*invA*	type III secretion system export apparatus protein InvA
*iroB*	salmochelin biosynthesis C-glycosyltransferase IroB
*iroC*	salmochelin/enterobactin export ABC transporter IroC
*lpfB*	long polar fimbrial chaperone LpfB
*lsrF*	3-hydroxy-5-phosphonooxypentane-2,4-dione thiolase LsrF
*mdsA*	multidrug efflux RND transporter periplasmic adaptor subunit MdsA
*mdsB*	multidrug efflux RND transporter permease subunit MdsB
mltE*	transglycosylase SLT domain-containing protein MltE
oafA*	O-antigen acetyltransferase OafA
*pagK*	vesicle-borne virulence factor PagK
*pagK*	vesicle-borne virulence factor PagK
*pagO*	PhoPQ-activated DMT family transporter PagO
pefA*	major pilin PefA
*pipA*	type III secretion system effector protease PipA
rcK*	complement resistance protein Rck
rfc*	O-antigen polymerase Rfc
rhuM*	cytoplasmic virulence protein RhuM
*ripR*	itaconate degradation transcriptional regulator RipR
safA*	Saf fimbria major subunit SafA
safB*	pili assembly chaperone SafB
*sarA*	anti-inflammatory response activator SarA
*sciR*	Shiga-like toxin A subunit SciR
*sinH*	intimin-like inverse autotransporter SinH
sodC1*	superoxide dismutase [Cu-Zn] SodC1
spvB*	SPI-2 type III secretion system effector NAD(+)--protein-arginine ADP-ribosyltransferase SpvB
spvD*	SPI-2 type III secretion system effector cysteine hydrolase SpvD
*sseI*	SPI-2 type III secretion system effector SseI
*sseK2*	type III secretion system effector arginine glycosyltransferase SseK2
*sseK3*	type III secretion system effector arginine glycosyltransferase SseK3
*sspH2*	SPI-2 type III secretion system effector E3 ubiquitin transferase SspH2
*stcA*	Stc fimbria major subunit StcA
*stcD*	Stc fimbria adhesin StcD
*stfA*	Stf fimbria major subunit StfA
*stiA*	Sti fimbria major subunit StiA
*stjB*	StjB fimbria biogenesis outer membrane usher protein StjB
*stm0854*	STM0854 family CoA ester lyase
*stm0859*	STM0859 family transcriptional regulator
stm2239*	antiterminator Q family protein
sugR*	AAA family ATPase SugR
traT*	conjugal transfer complement resistance protein TraT
*xis*	excisionase Xis
yigF*	inner membrane protein YigF

*denotes genes present in CFSAN000741 only.

Despite having short-read Illumina sequences available at NCBI, this inversion went undetected in these strains. To our knowledge, this inversion is the largest ever identified in *Salmonella*, surpassing the next largest known inversion of 1.62 Mbp found in *S.* Typhimurium [35]. While it has been noted that serovar Typhimurium tends to invert portions of its genome around ribosomal RNA (rRNA) regions, those segments are comparatively small, measuring only 70−80 kb, unlike the inversion observed in these isolates [36,37]. Furthermore, the flanking regions of this inversion do not appear to contain rRNA; instead, they are associated with a Gifsy-1 phage, which appears to have become grounded due to its inability to excise as evidenced by the absence of the anti-repressor gene in the prophage (S1 Table) [38]. With the *S.* Typhimurium inversion, the rRNA remain functional and the expression of the genes within the inversion remain intact, we did not investigate whether the inversion in Newport-1015 modified gene regulation in some way. Gammaproteobacteria have been shown to invert small sections of specific genes to phase variate in response to environment specific signals, and these variations are able to regulate both overall expression and modification of protein sequence [39]. Additionally, while these isolates were all collected from environmental sources, it is possible that the genetic inversion coupled with both the high sequence identity and presence of the flanking Gifsy-1 phage may be related to host-specific adaptations [40,41].

### Closely related *Salmonella* strains exhibit distinct pathogenicity dynamics in a live *C. elegans* host

**Fig 3:**
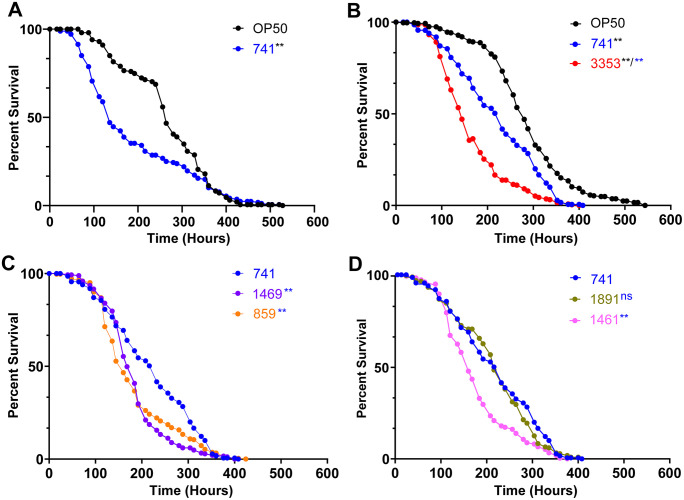
Impact of Salmonella strains on survival of C. elegans. **A:** S. Typhimurium (741, blue) shortens the survival of C. elegans adults compared to the normal diet of OP50 (black). **B:** C. elegans lifespan is shortened significantly more upon infection with CFSAN003353 (3353, red) compared to 741. **C:** CFSAN001469 (1469, purple) and CFSAN00859 (859, orange) caused greater reduction in worm lifespan compared to 741. **D:** Despite exhibiting the same PFGE pattern with the preserved inversion, strains CFSAN001461 (1461, pink) and CFSAN001891 (1891, olive) shortened worm lifespan to different degrees. 1461 caused significantly greater reduction than the control 741, whereas 1891 had the same magnitude of impact as 741. Data shown is combined from two independent trials (Table 2) and represents mean survival of the population in hours (m) ± SEM. P < 0.0001 (**). ns = not statistically significant. Colors of asterisks indicate the strain being used for comparison.

The *Salmonella* strains tested shortened worm survival as compared to animals on *E. coli* OP50. Importantly, these strains showed differential impacts on survival rates with significantly different time to death (TD_50_) for each, with trends that were similar between two independent trials (S2 Table). As a positive control, we exposed *C. elegans* to *S.* Typhimurium CFSAN000741 and found that, as previously reported, it shortens *C. elegans* mean survival by 15%−30% in independent trials (Fig 3A and Table 3) [18,19]. Upon testing the 6 strains used in this study, we found that the strongest, consistent pathogenic impact was seen upon exposure to CFSAN003353, which caused a significantly greater lifespan reduction as compared to *S*. Typhimurium (53% v 15%) (Fig 3B and Table 3). CFSAN001469 and CFSAN00859 also caused greater survival diminution compared to CFSAN000741, but their impacts were comparatively modest across trials (Table 3).

**Table 3:** Impact of *Salmonella* strains on *C. elegans* Survival.

Species	Strain ID	n = obs./total^a^	Mean Survival (Hours) ^a^	Std. Error (Hours) ^a^	p (v. OP50) ^a^	p (v. 741) ^a^
*E. coli*	OP50	246/296	284.37	6.12		0
*S.* Typhimurium	CFSAN000741	180/304	221.34	6.55	0	
*S.* Newport	CFSAN003353	206/253	166.20	4.48	0	0
*S.* Newport	CFSAN001469	248/292	185.62	4.17	0	5.80E-06
*S.* Newport	CFSAN000859	248/303	184.65	5.14	0	1.50E-03
*S.* Newport	CFSAN001891	215/267	218.91	5.56	0	1
*S.* Newport	CFSAN001461	272/310	176.30	4.40	0	1.20E-07

^a^Calculated from two independent trials.

Strikingly, we found that two of the strains, CFSAN001891 and CFSAN001461, which contain the large inversion and are > 99% identical to each other, exhibited distinctly different TD_50_s. In addition to the 15−30% shortening induced by CFSAN000741, CFSAN001461 exposure aggravated pathogen-driven survival diminution by an additional 25–30%, from approximately 236 hours to 150 hours, whereas CFSAN001891 had the same effect as CFSAN000741 (Figs 3C and 3D and Table 2). The underlying basis for the difference in virulence exhibited by the two strains, CFSAN001891 and CFSAN001461, is unclear from this study. However, these differences likely reflect variation in host–pathogen interactions driven by pathogen-specific features, host factors, or a combination of both, rather than a contradiction of their shared PFGE pattern or inversion status. In *C. elegans*, as in other species, virulence is determined not only by bacterial identity but also by how pathogen-associated features engage host immune pathways. Subtle differences in bacterial surface structures or secreted factors could differentially activate or evade host defense mechanisms, resulting in divergent survival outcomes even among closely related strains. Conversely, host-intrinsic factors may also contribute to strain-specific responses, including genetic variation that alters how the host perceives or responds to distinct pathogen strains. Consistent with this idea, strain-specific host responses to highly related pathogens such as *Enterococcus faecalis* and Bacillus thuringiensis (Bt) have been previously reported [42,43]. Notably, transcription factors AP-1 and ELT-2 have been implicated in mediating shared versus strain-specific host responses to Bt, respectively [43]. Future studies, such as tandem host–pathogen RNA sequencing, will be essential to elucidate the molecular mechanisms underlying the virulence differences observed here.

## Conclusion

The *C. elegans* model has proven to be an invaluable tool for assessing a phenotype that might otherwise have gone unnoticed. Together, the experiments presented here underscored the feasibility of using *C. elegans* to measure the virulence capacity of environmental *Salmonella* strains as well as nuanced pathogenicity differences likely resulting from genomic events and their downstream host interactions. Given that there were fewer than ten confirmed illnesses linked to Newport-1015 (<10 from 2000−2018), the virulence phenotype discovered in this study is surprising and suggests that this genomovar may not be preferentially adapted to human hosts though sharing high sequence identity with strains known to have caused large illness outbreaks. The isolates studied here offer a unique opportunity to elucidate the underlying regulatory mechanisms that may contribute to not only the differences in virulence among *Salmonella* serovars but also host adaptive genetic markers. Moreover, due to the highly conserved nature of the genomes, distinguishing differences between the two genomovars (Newport-61 vs. Newport-1015) can currently only be achieved using PFGE or long-read sequencing. Comparative genomics alone is insufficient in drawing conclusions about the virulence potential of closely related strains of *Salmonella*. For this reason, expression studies will be required to identify the mechanisms underlying these phenotypes, along with genome-wide screening to understand the roles that specific genes play in the infection process.

## Supporting information

S1 Table Prophages and prophage-like elements in the *Salmonella* strains from these experiments. (XLSX)

S2 Table Impact of *Salmonella* strains on *C. elegans* Survival (Independent Trials). (XLSX)

## References

[1] Dewey-Mattia D, Kisselburgh H, Manikonda K, Silver R, Subramhanya S, Sundararaman P, et al. Surveillance for foodborne disease outbreaks–United States, 2016: annual report; 2018. 10.15585/mmwr.ss6710a1 PMC6061962 30048426

[2] Bishop R, Erdman M, Fields P, Fullerton K, Jackson K, Mahon B. National enteric disease surveillance: Salmonella surveillance overview. Georgia, USA: National Center for Emerging and Zoonotic Infectious Diseases; 2011.

[3] CDC CfDCaP. BEAM (Bacteria, Enterics, Ameba, and Mycotics) Dashboard Atlanta, Georgia: US Department of Health and Human Services; 2025. Available from: www.cdc.gov/ncezid/dfwed/BEAM-dashboard.html

[4] Greene SK, Daly ER, Talbot EA, Demma LJ, Holzbauer S, Patel NJ, et al. Recurrent multistate outbreak of Salmonella Newport associated with tomatoes from contaminated fields, 2005. Epidemiol Infect. 2008;136(2):157–65. doi: 10.1017/S095026880700859X 17475091 PMC2870807

[5] Hernández-Reyes C, Schikora A. Salmonella, a cross-kingdom pathogen infecting humans and plants. FEMS Microbiol Lett. 2013;343(1):1–7. doi: 10.1111/1574-6968.12127 23488473

[6] CDC CfDCaP. National enteric disease surveillance: Salmonella annual report, 2016; 2016.

[7] Angelo KM, Chu A, Anand M, Nguyen T-A, Bottichio L, Wise M, et al. Outbreak of Salmonella Newport infections linked to cucumbers--United States, 2014. MMWR Morb Mortal Wkly Rep. 2015;64(6):144–7. 25695319 PMC4584703

[8] Whitney BM, McClure M, Hassan R, Pomeroy M, Seelman SL, Singleton LN, et al. A series of papaya-associated Salmonella illness outbreak investigations in 2017 and 2019: a focus on traceback, laboratory, and collaborative efforts. J Food Prot. 2021;84(11):2002–19. doi: 10.4315/JFP-21-082 34265065

[9] FDA UFaDA. Factors potentially contributing to the contamination of red onions implicated in the summer 2020 outbreak of Salmonella Newport. 2021.

[10] Ottesen A, Ramachandran P, Reed E, Gu G, Gorham S, Ducharme D, et al. Metagenome tracking biogeographic agroecology: phytobiota of tomatoes from Virginia, Maryland, North Carolina and California. Food Microbiol. 2019;79:132–6. doi: 10.1016/j.fm.2018.12.001 30621868

[11] Crim SM, Chai SJ, Karp BE, Judd MC, Reynolds J, Swanson KC, et al. Salmonella enterica serotype Newport infections in the United States, 2004–2013: increased incidence investigated through four surveillance systems. Foodborne Pathog Dis. 2018;15(10):612–20. 30036085 10.1089/fpd.2018.2450 PMC6263033

[12] Tran TD, Luallen RJ. An organismal understanding of C. elegans innate immune responses, from pathogen recognition to multigenerational resistance. Semin Cell Dev Biol. 2024;154(Pt A):77–84. doi: 10.1016/j.semcdb.2023.03.005 36966075 PMC10517082

[13] Aballay A, Ausubel FM. Caenorhabditis elegans as a host for the study of host-pathogen interactions. Curr Opin Microbiol. 2002;5(1):97–101. doi: 10.1016/s1369-5274(02)00293-x 11834377

[14] Harding BW, Ewbank JJ. An integrated view of innate immune mechanisms in C. elegans. Biochem Soc Trans. 2021;49(5):2307–17. doi: 10.1042/BST20210399 34623403

[15] Kim DH. Signaling in the innate immune response; 2015. 10.1895/wormbook.1.83.2 PMC6369418 26694508

[16] Peterson ND, Pukkila-Worley R. Caenorhabditis elegans in high-throughput screens for anti-infective compounds. Curr Opin Immunol. 2018;54:59–65. doi: 10.1016/j.coi.2018.06.003 29935375 PMC6463281

[17] Sifri CD, Begun J, Ausubel FM. The worm has turned--microbial virulence modeled in Caenorhabditis elegans. Trends Microbiol. 2005;13(3):119–27. doi: 10.1016/j.tim.2005.01.003 15737730

[18] Aballay A, Yorgey P, Ausubel FM. Salmonella typhimurium proliferates and establishes a persistent infection in the intestine of *Caenorhabditis elegans*. Curr Biol. 2000;10(23):1539–42. doi: 10.1016/s0960-9822(00)00830-7 11114525

[19] Labrousse A, Chauvet S, Couillault C, Kurz CL, Ewbank JJ. Caenorhabditis elegans is a model host for Salmonella typhimurium. Curr Biol. 2000;10(23):1543–5. doi: 10.1016/s0960-9822(00)00833-2 11114526

[20] Bell RL, Zheng J, Burrows E, Allard S, Wang CY, Keys CE, et al. Ecological prevalence, genetic diversity, and epidemiological aspects of Salmonella isolated from tomato agricultural regions of the Virginia Eastern Shore. Front Microbiol. 2015;6:415. doi: 10.3389/fmicb.2015.00415 25999938 PMC4423467

[21] Gu G, Strawn LK, Oryang DO, Zheng J, Reed EA, Ottesen AR, et al. Agricultural practices influence Salmonella contamination and survival in pre-harvest tomato production. Front Microbiol. 2018;9:2451. doi: 10.3389/fmicb.2018.02451 30386314 PMC6198144

[22] Ferreira CM, Jang JH, Hoffmann M, Lou Y, Literman R, Brown EW, et al. Closed genome sequences of 14 Salmonella enterica serovar Newport isolates from various sources. Microbiol Resour Announc. 2025;14(4):e0079624. doi: 10.1128/mra.00796-24 40079577 PMC11984164

[23] Becker D, Selbach M, Rollenhagen C, Ballmaier M, Meyer TF, Mann M, et al. Robust Salmonella metabolism limits possibilities for new antimicrobials. Nature. 2006;440(7082):303–7. doi: 10.1038/nature04616 16541065

[24] Ferreira CM, Jang JH, Hoffmann M, Lou Y, Literman R, Brown EW, et al. Closed genome sequences of 14 Salmonella enterica serovar Newport isolates from various sources. Microbiol Resour Announc. 2025;14(4):e0079624. doi: 10.1128/mra.00796-24 40079577 PMC11984164

[25] Tatusova T, DiCuccio M, Badretdin A, Chetvernin V, Nawrocki EP, Zaslavsky L, et al. NCBI prokaryotic genome annotation pipeline. Nucleic Acids Res. 2016;44(14):6614–24. doi: 10.1093/nar/gkw569 27342282 PMC5001611

[26] Page AJ, Cummins CA, Hunt M, Wong VK, Reuter S, Holden MTG, et al. Roary: rapid large-scale prokaryote pan genome analysis. Bioinformatics. 2015;31(22):3691–3. doi: 10.1093/bioinformatics/btv421 26198102 PMC4817141

[27] Hadfield J, Croucher NJ, Goater RJ, Abudahab K, Aanensen DM, Harris SR. Phandango: an interactive viewer for bacterial population genomics. Bioinformatics. 2018;34(2):292–3. doi: 10.1093/bioinformatics/btx610 29028899 PMC5860215

[28] Wishart DS, Han S, Saha S, Oler E, Peters H, Grant JR, et al. PHASTEST: faster than PHASTER, better than PHAST. Nucleic Acids Res. 2023;51(W1):W443–50. doi: 10.1093/nar/gkad382 37194694 PMC10320120

[29] Roer L, Hendriksen RS, Leekitcharoenphon P, Lukjancenko O, Kaas RS, Hasman H, et al. Is the evolution of Salmonella enterica subsp. enterica Linked to Restriction-Modification Systems? mSystems. 2016;1(3):e00009-16. doi: 10.1128/mSystems.00009-16 27822532 PMC5069764

[30] Feldgarden M, Brover V, Gonzalez-Escalona N, Frye JG, Haendiges J, Haft DH, et al. AMRFinderPlus and the Reference Gene Catalog facilitate examination of the genomic links among antimicrobial resistance, stress response, and virulence. Sci Rep. 2021;11(1):12728. doi: 10.1038/s41598-021-91456-0 34135355 PMC8208984

[31] Johnson M, Zaretskaya I, Raytselis Y, Merezhuk Y, McGinnis S, Madden TL. NCBI BLAST: a better web interface. Nucleic Acids Res. 2008;36(Web Server issue):W5-9. doi: 10.1093/nar/gkn201 18440982 PMC2447716

[32] Amrit FRG, Ratnappan R, Keith SA, Ghazi A. The C. elegans lifespan assay toolkit. Methods. 2014;68(3):465–75. doi: 10.1016/j.ymeth.2014.04.002 24727064

[33] Keith SA, Amrit FRG, Ratnappan R, Ghazi A. The C. elegans healthspan and stress-resistance assay toolkit. Methods. 2014;68(3):476–86. doi: 10.1016/j.ymeth.2014.04.003 24727065

[34] Han SK, Lee D, Lee H, Kim D, Son HG, Yang J-S, et al. OASIS 2: online application for survival analysis 2 with features for the analysis of maximal lifespan and healthspan in aging research. Oncotarget. 2016;7(35):56147–52. doi: 10.18632/oncotarget.11269 27528229 PMC5302902

[35] Krug PJ, Gileski AZ, Code RJ, Torjussen A, Schmid MB. Endpoint bias in large Tn10-catalyzed inversions in Salmonella typhimurium. Genetics. 1994;136(3):747–56. doi: 10.1093/genetics/136.3.747 8005430 PMC1205881

[36] Liu SL, Sanderson KE. Homologous recombination between rrn operons rearranges the chromosome in host-specialized species of Salmonella. FEMS Microbiol Lett. 1998;164(2):275–81. doi: 10.1111/j.1574-6968.1998.tb13098.x 9682477

[37] Lehner AF, Harvey S, Hill CW. Mapping and spacer identification of rRNA operons of Salmonella typhimurium. J Bacteriol. 1984;160(2):682–6. doi: 10.1128/jb.160.2.682-686.1984 6094483 PMC214789

[38] Ramisetty BCM, Sudhakari PA. Bacterial “grounded” prophages: hotspots for genetic renovation and innovation. Front Genet. 2019;10:65. doi: 10.3389/fgene.2019.00065 30809245 PMC6379469

[39] Chanin RB, West PT, Wirbel J, Gill MO, Green GZM, Park RM, et al. Intragenic DNA inversions expand bacterial coding capacity. Nature. 2024;634(8032):234–42. doi: 10.1038/s41586-024-07970-4 39322669

[40] Matthews TD, Rabsch W, Maloy S. Chromosomal rearrangements in Salmonella enterica serovar Typhi strains isolated from asymptomatic human carriers. mBio. 2011;2(3):e00060-11. doi: 10.1128/mBio.00060-11 21652779 PMC3107234

[41] Carasso S, Keshet-David R, Zhang J, Hajjo H, Kadosh-Kariti D, Gefen T, et al. Bacteriophage-driven DNA inversions shape bacterial functionality and long-term co-existence in Bacteroides fragilis. Gut Microbes. 2025;17(1):2501492. doi: 10.1080/19490976.2025.2501492 40350564 PMC12068327

[42] Garsin DA, Sifri CD, Mylonakis E, Qin X, Singh KV, Murray BE, et al. A simple model host for identifying Gram-positive virulence factors. Proc Natl Acad Sci U S A. 2001;98(19):10892–7. doi: 10.1073/pnas.191378698 11535834 PMC58570

[43] Zárate-Potes A, Yang W, Pees B, Schalkowski R, Segler P, Andresen B, et al. The C. elegans GATA transcription factor elt-2 mediates distinct transcriptional responses and opposite infection outcomes towards different Bacillus thuringiensis strains. PLoS Pathog. 2020;16(9):e1008826. doi: 10.1371/journal.ppat.1008826 32970778 PMC7513999

