# Correction: Evolving threats: Leveraging *C. elegans* to decode the virulence profiles of highly related environmental *Salmonella* Newport isolates

**DOI:** 10.1371/journal.pone.0351322

**Published:** 2026-06-09

**Authors:** Christina M. Ferreira, Mirae Choe, Bella Wayhs, Julie A. Haendiges, Robert Literman, Jianghong Meng, Arjuman Ghazi, Rebecca L. Bell

The seventh author’s name is spelled incorrectly. The correct name is: Arjumand Ghazi.

The images for Figs 1 and 2 are incorrectly switched. The image that appears as Fig 1 should be Fig 2, and the image that appears as Fig 2 should be Fig 1. The figure captions appear in the correct order. The authors have provided a corrected version of the figures here.

The specie name *C. elegans* and ‘*S.*’ should be italicized in the caption of Fig 3 and throughout the article. Please see the correct Fig 3 here.

**Fig 1:**
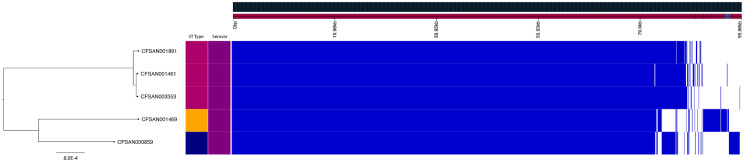
Phylogeny showing gene presence/absence of key Salmonella Newport isolates. The phylogenetic tree shows the relatedness of the S. Newport isolates to one another. The blue colored area represents the presence/absence of genes across the entirety of the genomes. The sequence types represented include 118 (magenta), 350 (yellow) and 5 (dark blue).

**Fig 2:**
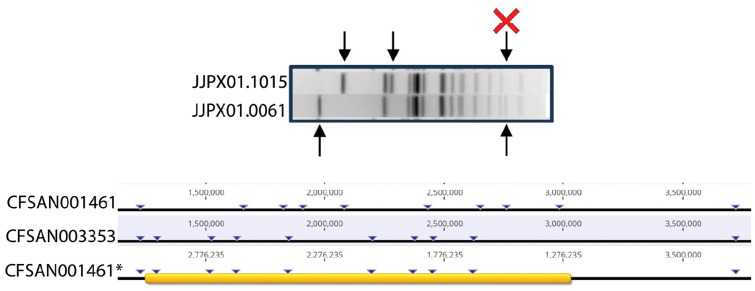
Inversion confirmation of CFSAN001461 and CFSAN1891 using in silico PFGE analysis. PFGE *XbaI* pattern of JJPX01.1015 compared to JJPX01.0061 (top), where the arrows highlight the differing bands in the PFGE patterns and the red “X” denotes the band that is not present in the 1015. The inversion identified in JJPX01.1015 accounts for the differences in the PFGE patterns due to changes in cut site locations within the genome. The locations of these cut site changes (bottom) due to the directionality of the inversion – by manually rotating the 1.7Mbp JJPX01.1015 inversion at the Gifsy-1 sites (denoted by CFSAN001461*) and subsequently performing *in silico* digestion with *XbaI*, the cut sites revert to the same fragment sizes reflected in JJPX01.0061 (CFSAN003353) confirming the accuracy of inversion in the closed genome.

**Fig 3:**
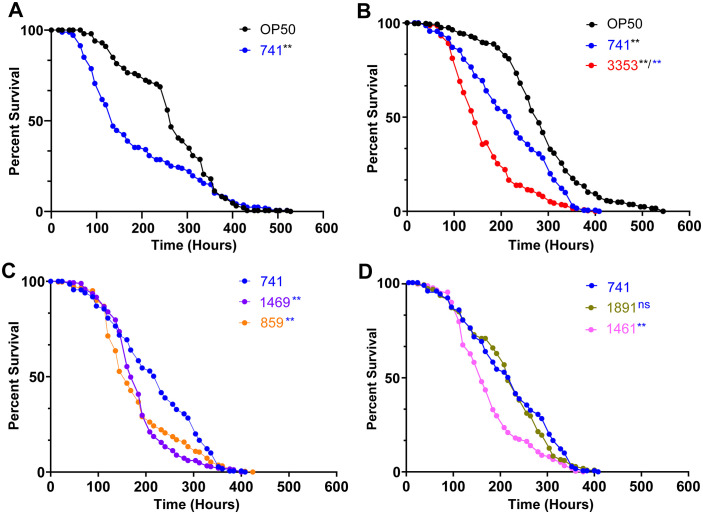
Impact of Salmonella strains on survival of *C. elegans.* **A:** S. Typhimurium (741, blue) shortens the survival of *C. elegans* adults compared to the normal diet of OP50 (black). **B:** *C. elegans* lifespan is shortened significantly more upon infection with CFSAN003353 (3353, red) compared to 741. **C:** CFSAN001469 (1469, purple) and CFSAN00859 (859, orange) caused greater reduction in worm lifespan compared to 741. **D:** Despite exhibiting the same PFGE pattern with the preserved inversion, strains CFSAN001461 (1461, pink) and CFSAN001891 (1891, olive) shortened worm lifespan to different degrees. 1461 caused significantly greater reduction than the control 741, whereas 1891 had the same magnitude of impact as 741. Data shown is combined from two independent trials (Table 2‌‌) and represents mean survival of the population in hours (m) ± SEM. P < 0.0001 (**). ns = not statistically significant. Colors of asterisks indicate the strain being used for comparison.
